# A Review of Cardiogenic Shock in Acute Myocardial Infarction

**DOI:** 10.2174/157340308783565456

**Published:** 2008-02

**Authors:** L Khalid, S.H Dhakam

**Affiliations:** Department of Medicine, Aga Khan University, Stadium Road, P.O. Box 3500, Karachi, Pakistan

**Keywords:** Cardiogenic shock, fibrinolytic therapy, percutaneous coronary intervention, myocardial infarction, intra-aortic balloon counterpulsation, coronary artery bypass grafting.

## Abstract

Cardiogenic shock continues to be the most common cause of death in patients hospitalized with acute myocardial infarction. It has also been frequently associated with ST-segment elevation myocardial infarction (STEMI) and patients with co-morbidities. Cardiogenic shock presents with low systolic blood pressure and clinical signs of hypoperfusion. Rapid diagnosis and supportive therapy in the form of medications, airway support and intra-aortic balloon counterpulsation is required. Initial stabilization can be followed by reperfusion by fibrinolytic therapy, emergent percutaneous intervention (PCI) or coronary artery bypass grafting (CABG). The latter two have been found to decrease mortality in the long term. Research is being carried out on the role of inflammatory mediators in the clinical manifestation of cardiogenic shock. Mechanical support devices also show promise in the future.

## INTRODUCTION

Cardiogenic shock is the most common cause of death in patients with acute myocardial infarction (AMI) [1-9] and has a frequency of around 7-10% [1, 2, 10]. It continues to cause significant mortality despite advances in pharmacological, mechanical and reperfusion endeavors.

## DEFINITION

Cardiogenic shock is defined as a systolic blood pressure of less than 90 mmHg for at least 30 minutes, which is secondary to myocardial dysfunction. It is associated with clinical signs of hypoperfusion, which include decreased urine output, altered mental status and peripheral vasoconstriction. It is usually unresponsive to fluids, an important differentiating quality from other types of shock. However, it frequently responds to inotropes. The cardiac index (CI) and the pulmonary capillary wedge pressure (PCWP) are usually less than 2.2 l/min/m^2^ and greater than 15 mmHg respectively [11].

## INCIDENCE

The incidence within the community over a 23-year period (1975-1997) was found to be 7.1% [6]. In the, Global Utilization of Streptokinase and Tissue Plasminogen Activator for Occluded Arteries (GUSTO-1) trial [7], the incidence of cardiogenic shock was likewise 7.2%, and consistent with other studies [12-14]. However, it is difficult to assess the actual number, as a certain proportion of patients die before reaching the hospital and cannot be categorized as having cardiogenic shock [2, 12, 14]. Over the years, there has been little decrease in the time to present to the hospital [15].

A larger number of patients develop shock after reaching the hospital. This highlights an important fact that medical contact may have been established before shock development and opens the door to its possible prevention. In the GUSTO trial, 11% of patients had shock on presentation while 89% of patients subsequently developed shock [7]. Similarly in the SHould we emergently revascularise Occluded Coronaries for cardiogenic shock? (SHOCK) trial registry, more than half the patients developed shock within a day of presenting to the hospital [4]. Early shock, defined as occurring in less than 24 hours, was found in 74.1% of patients in a recent study [16]. In addition, it has been observed that there is a slight increase in the number of deaths among patients who present with early shock [17].

Despite emerging innovative treatments, in-hospital mortality in patients with cardiogenic shock continues to be as high as 70-80% [1, 2]. Other studies have quoted mortality rates of around 50% to 80% [4]. A study carried out at a tertiary care hospital in Pakistan had an in-hospital mortality rate of 55% [18]. In another study, the overall in-hospital mortality was high (63%) but was found to reduce (P=0.004) over time from 1992 to 1997. This was partially attributed to the greater use of revascularization procedures, which are known to improve outcomes [19].

Cardiogenic shock seems to occur with a greater frequency amongst patients with ST-segment elevation myocardial infarction (STEMI). It was observed that shock developed in 7.5% of patients with STEMI [2, 7] and in 2.5% of patients with non-ST-segment elevation myocardial infarction (NSTEMI) [20]. In another study, 4.2% of patients with STEMI and 2.5% of patients with NSTEMI had cardiogenic shock. A significant delay precedes shock development in patients with NSTEMI [21]. The underlying reason may be the rapid cell necrosis that takes place in STEMI contrasting with a slower cell loss in NSTEMI. Thus, the highest creatine kinase (CK) level is found in STEMI as compared to NSTEMI [20].

Diabetics are twice as likely to develop cardiogenic shock as non-diabetics with AMI. However, the prognosis of cardiogenic shock is similar in both groups of patients [22].

## ETIOLOGY

Left ventricular dysfunction (LVD) is the most frequent cause of cardiogenic shock [23]. In a recent trial, it was highlighted that LVD was the main etiology [4, 24], occurring in 74.5% of patients. This was followed by acute mitral regurgitation (8.3%), ventricular septal rupture (4.6%), isolated right ventricular shock (3.4%), tamponade or cardiac rupture (1.7%), and other causes (8%) (Table **1**). Infarctions were located anteriorly in most of the patients (55%) in the SHOCK trial registry [4, 24]. 	While 46% of the infarctions were inferior, 21% were posterior, and 50% were in multiple locations. Other studies have found similar results [25]. Around 60% of patients had triple vessel disease while left main disease was encountered in 20% [26]. The left anterior descending artery (LAD) was found to be the most frequently involved artery unrelated to the time of shock onset [17]. Thus, severe arterial disease precedes shock development.

The median time from the onset of infarction to shock development was 5.6 hours [4]. In the SHOCK trial registry it was found to be 6.2 hr [16]. Certain characteristics (Table **2**) such as, being elderly, diabetic or having anterior infarction predispose patients with myocardial infarction to develop shock [1, 27-29]. Some other factors include presence of previous infarction, peripheral vascular disease, cerebrovascular disease, reduced ejection fractions and larger infarctions [27-28]. Age was most strongly associated with shock. In another study, age, systolic blood pressure, heart rate and Killip class at presentation contributed to more than 85% of the predictive data for increased risk of shock [30]. Clinicians should be more vigilant in such cases and monitor these patients more frequently and aggressively.

Cardiac power was predicated to be the strongest independent hemodynamic factor associated with mortality in patients admitted to hospitals with cardiogenic shock. It was also observed that older age and females had reduced cardiac power [31].

## PATHOPHYSIOLOGY

Ischemia due to decreased coronary perfusion leads to muscle hypoxia and necrosis which compromises myocardial contractility. This leads to decreased cardiac output and a subsequent drop in the arterial blood pressure. Simultaneously, the body’s sympathetic system responds to the reduced blood pressure by increasing vasoconstriction. The hormonal system is also activated leading to salt and water retention. This has a detrimental role, as the coronary perfusion is further compromised. A vicious cycle is thus created and it leads to decreased perfusion at the tissue level. Lactic acidosis and hypoxia eventually sets in, which further compromise the myocardial contractility until the arterial blood pressure is not maintained to a level needed to sustain life.

## MANAGEMENT

### Assessment

Cardiogenic shock is an emergency and it needs rapid diagnosis and institution of therapy. Improved long-term outcomes require immediate diagnosis and management and if needed, transfer to a tertiary care hospital [32].

History will usually reveal symptoms of a preceding AMI. A diagnosis of cardiogenic shock is made when myocardial dysfunction is observed in the absence of other causes such as hemorrhage, sepsis, pulmonary embolism, tamponade, aortic dissection, and preexisting valvular disease [32]. On a physical exam (Table **3**), the patient may be cyanotic with cold extremities and pulses are usually rapid and faint. If LVD is the etiology, then jugular venous distention (JVD) and rales in the lung field due to pulmonary congestion are observed. If right ventricular failure is the underlying cause then JVD and kussmaul’s sign is present and pulmonary rales are not found. Other findings include distant heart sounds and the presence of third and fourth heart sounds. Mitral regurgitation or a VSD can lead to a new systolic murmur. Arrhythmias are a frequent occurrence and need immediate attention.

64% of patients in the SHOCK trial registry, presented with hypotension and signs of hypoperfusion such as tachycardia, altered sensorium, decreased urine output and cool extremities. These patients also had signs of pulmonary congestion [33]. A smaller proportion of patients (28%) experienced shock with no pulmonary congestion, which is also known as the silent lung syndrome. However, clinicians should be wary and refrain from incorrectly associating absence of pulmonary congestion with decreased risk [33]. Thus, signs of hypoperfusion alone are mortality indicators regardless of the presence or absence of pulmonary congestion [33]. Similarly, the GUSTO-I study observed increased mortality at 30 days if oliguria, cold extremities or altered sensorium were found to be present [34]. Another study also ascertained oliguria and cold extremities as independent and strong mortality predictors [20]. Thus, the initial clinical assessment of a patient is important in determining the future prognosis.

### Diagnosis

Tools that facilitate diagnosis (Table **4**) include an electrocardiogram (ECG), cardiac enzymes, chest X ray, arterial blood gases (ABG), electrolytes, complete blood count (CBC) and/or a coagulation profile [35]. Invasive hemodynamic monitoring may be required but is not a necessity. Bedside echocardiography plays a vital role in assessing the possible contributing causes [36, 37]. It can be used to quickly rule out or diagnose mitral regurgitation, ventricular septal or free wall tear, tamponade or a pericardial effusion so that emergent intervention can be instituted.

There is controversial evidence regarding right heart catheterization. One study claimed improved outcomes [38] by using this invasive procedure while increased mortality was observed in a different study [39].

## TREATMENT

### Initial Stabilization

Aspirin and heparin constitute the first line of treatment. Fluids may need to be given in order to rule out hypovolemic shock. These need to be instituted with continuous monitoring of clinical signs, such as urine output, blood pressure and heart rate. In right ventricular failure, fluid support is required and nitrates and morphine should be avoided, as they tend to increase hypotension. Oxygen should be given through a face mask or if the need arises, airway should be secured and mechanical ventilation started. Arrhythmias are a frequent occurrence and sustained tachyarrhythmias need to be converted electrically so as to avoid further compromise of the cardiac output. Bradyarrhythmias may require atropine or temporary pacing [35].

Morphine reduces sympathetic stimulation and should be given for pain relief. It also decreases the preload and decompresses the left ventricle [35]. Nitroglycerine, a venodilator, has limited use in cardiogenic shock. It is important to maintain the mean arterial blood pressure above 90 mmHg. For this purpose, inotropes and vasopressors like dopamine and catecholamines may be required. Dopamine, an inotrope and a vasopressor, is preferred initially. Another alternative is dobutamine but its use may produce vasodilation and lead to hypotension [32]. In some situations, a combination of dopamine and dobutamine are more beneficial than the use of either agent alone [40]. Persistently low systolic blood pressures with values such as 70 mmHg require addition of more drugs such as norepinephrine. Pressor agents should be used carefully as they increase the heart rate and may trigger arrythmias. Diuretics are added if pulmonary congestion is present to help in increasing the oxygenation [32]. Electrolyte imbalances and metabolic acidosis need immediate treatment. Nitrates, b-blockers, and angiotensin-converting enzyme inhibitors help to improve outcomes after myocardial infarction [41]. However, beta-blockers can deteriorate the condition and are generally avoided in shock.

## SUPPORTIVE MEASURES

### Intra-Aortic Balloon Pump (IABP)

Intra-aortic balloon pump (IABP) is required for stabilizing patients before reperfusion therapies. It increases coronary blood flow during diastole and decreases the afterload by lowering the systemic vascular resistance during systole. However, if there is severe coronary stenosis, there is little improvement in coronary perfusion [42]. The GUSTO-1 trial [43], observed a decreased mortality rate at 30 days in patients treated with IABP (46%) as compared to those who did not receive this treatment (60%, P = 0.11). Similarly, in the SHOCK trial, patients who received treatment with IABP had reduced rates of mortality (50%) within hospital, versus those who did not have IABP placement (72%, P<0.0001) [44]. It was used in both the arms of the study: medical management and revascularisation.

The American College of Cardiology/American Heart Association (ACC/AHA) STEMI guidelines [45] give a class I recommendation for use of IABP when cardiogenic shock cannot be managed on medications alone. This allows stabilization for angiography and revascularization procedures.

Thus, in hospitals where emergent revascularization is not available, it is more appropriate to proceed with fibrinolytic therapy and IABP while arrangements are made for percutaneous transluminal coronary angioplasty (PTCA) or coronary artery bypass graft (CABG). This might be a more realistic approach in many developing countries like Pakistan where there may be unavailability of immediate revascularization facilities in hospitals.

### Reperfusion Strategies

#### Fibrinolytic Therapy

Fibrinolytic therapy, when used in patients with AMI without shock, decreases shock onset [7, 46-49]. This is a vital fact, as it is known that shock development occurs after six hours of presentation to the hospital [4, 7, 13, 24]. These six hours are crucial for institution of treatment and can play a role in the prevention of cardiogenic shock.

In some trials, outcomes have been found to be similar with the use of streptokinase versus placebo and between streptokinase and tissue plasminogen activator [7, 12, 13]. However, in the SHOCK registry, patients receiving fibrinolytic therapy had lower mortality rates (54%) in the hospital versus those who did not receive fibrinolytic therapy (64%, P=0.005) [44]. But confounding factors may have been involved.

The outcome of treatment with fibrinolytics is associated with the degree of reperfusion [50, 51] but in patients with cardiogenic shock it is reduced [25, 51, 52] due to decreased coronary perfusion. In conclusion, the use of fibrinolytics does not increase survival in patients with an ongoing cardiogenic shock.

Hospitals lacking revascularization capabilities may pursue IABP and fibrinolytic therapy while making arrangements to transfer to hospitals with revascularisation capabilities [44].

#### Percutaneous Coronary Intervention

In the GUSTO-1 trial, a reduced mortality rate (43%) was observed at 30 days with successful PTCA as compared to those without PTCA (61%) as a mode of treatment [21]. The Swiss Multicenter Trial of Angioplasty Shock (SMASH) trial [53, 54] also showed decreased mortality rates in patients treated invasively rather than medically. However, it did not reach statistical significance. The SHOCK trial [5] also compared emergent revascularization versus immediate medical stabilization. The latter involved use of fibrinolytic therapy, inotropic and vasopressor agents. IABP was used quite frequently (86%) in patients. The revascularization arm was further dichotomized: 64% of patients underwent PCI and 36% had coronary artery bypass surgery. However, the mortality rates in the two groups of revascularization and medical treatment were not found to be statistically significant at 30 days (46.7% *vs*. 56.0%, respectively, P = 0.11), but by 6-months there was some divergence and significantly increased survival rates were observed in patients treated with revascularization (50.3% *vs*. 63.1%, P=0.027). Thus long-term benefits emerged subsequently and a significant mortality reduction was observed at 6 months consistent with 13 lives saved per 100 patients treated [5]. Diabetics have similar improved outcomes as non-diabetics, if revascularization is used, as observed in both the SHOCK Trial Registry and the SHOCK trial [55]. Furthermore, after one year, survival was 46.7% for patients in the revasularisation group compared with 33.6% in the medically treated group (P<0.03) [56]. Thus, significant long-term mortality benefits are observed with emergent revascularisation. Consistent with the above data, mortality due to cardiogenic shock in AMI has been found to be lower in hospitals with catheterization facilities than in hospitals without such facilities [57].

Analysis of the subgroup constituting patients less than 75 years, underscores a marked interaction between patient age and treatment outcome. A decrease in mortality by 15 % at 30 days is observed in patients less than 75 years of age treated by revascularisation. At 6 months, the reduction in mortality was found to be 20%. However, in patients 75 years and above an increase in mortality by 22% was observed when patients were treated with revascularization rather than initial medical stabilization [5]. 

The outcome of PTCA is important in determining the survival of patients. The degree of reperfusion in the infarct related artery is associated with outcomes [58]. In the recent SHOCK trial, it was observed that the 30-day mortality rate was reduced with successful angioplasty (38%) as compared to patients with unsuccessful angioplasty (79%) [5]. However, reperfusion is lower in patients with cardiogenic shock [59, 60]. Patients with shock have comparatively less successful reperfusion rates (54% to 100%) with PTCA in the infarct-causing artery, than in patients without shock [61-71].

There are some indications that stents are an important ancillary part of cardiogenic shock management. [72]. In the Platelet Glycoprotein IIb/IIIa in Unstable Angina: Receptor Suppression Using Integrilin Therapy (PURSUIT) trial, use of eptifibatide had no consequence on the development of shock, in patients with non-ST elevation acute coronary syndromes, but a mortality reduction from 73.5% to 58.5% (P=0.03) was seen [73]. Lack of stent and glycoprotein IIb/IIIa usage has been observed as predictors of mortality in a recent study. Six predictors of mortality in patients presenting with shock included age, female gender, creatinine levels, total blockage of the left anterior descending artery (LAD), absence of stent use, absence of glycoprotein IIb/IIIa inhibitor use, during PCI. These were all found to be statistically significant. A second analysis carried out with variables identified at the time of initial presentation found gender, age, renal insufficiency, and total occlusion of the left anterior descending coronary artery to be significant [74].

Thus, in conclusion, the current American College of Cardiology/American Heart Association (ACC/AHA) guidelines recommend (class I) early revascularisation strategy for patients < 75 years of age with cardiogenic shock [75]. It is also recommended that patients especially less than 75 years of age should be promptly transferred to tertiary care hospitals where revascularization can be performed [56].

#### Surgical Intervention

Patients in cardiogenic shock undergoing emergent coronary artery bypass surgery have mortality rates of around 25% to 60% [76]. In the SHOCK trial, 36% of the patients randomized to revascularization in that study were treated surgically [5]. Such patients were more likely to have left main disease, 3-vessel disease and diabetes than those treated with PCI. However, similar survival rates were observed in the two groups: 55.6% in the PCI group compared with 57.4% in the CABG group at 30 days (P=0.86). Likewise after one year, the rates were 51.9% (PCI) versus 46.8% (CABG), respectively, (P=0.71). [77].

Some retrospective studies quote improved outcomes when CABG is used as an emergency procedure for AMI and cardiogenic shock [78-81]. In patients with left main or triple-vessel disease, stabilization by IABP and immediate activation of the surgery team should be sought, as CABG may be the more desired procedure in such patients to establish complete revascularization [5].

Patients presenting with additional mechanical complications such as acute mitral regurgitation due to papillary muscle rupture or LV free wall or septal rupture need surgery for survival, however, the outcome in such patients is much worse [35]. Ventricular septal rupture has a high in-hospital mortality of around 87% as observed in the SHOCK registry. Patients undergoing surgery for ventricular septal rupture have a survival rate of 19% [82].

Diagnosis of shock heralds a high mortality rate of around 50%, regardless of the benefits of early PCI or CABG. Around 50% of these deaths occur in the first 2 days [23, 83].

### Other Mechanical Supports

IABP is useful in providing mechanical support during shock. It has been observed that cardiac index can indicate if potential benefit can be derived from IABP. The use of IABP has better outcomes when used in patients with a cardiac index higher than 1.2 L/min/m^2^ and a systemic vascular resistance less than 2100 dynes/sec/cm^–5^. However, if the cardiac index is lower than 1.2 L/min/m^2^, outcome is poor even if IABP is used. In such cases other support devices may be required [84].

Extracorporeal life support (ECLS) has been utilized in cases of severe cardiac or pulmonary failure [85]. Percutaneous cardiopulmonary bypass can also provide support and can be performed at the bedside through the femoral artery and vein [86]. These two devices however, do not help in unloading the left ventricle.

Biventricular assist devices, can serve as a bridge to cardiac transplantation. This has been found to have a success rate of around 59% [87]. Experimental percutaneous LV assist devices help to unload the left ventricle [88] and present another option.

### New Pharmacological Agents

During the development of cardiogenic shock, the body may launch a systemic inflammatory response, which has led to investigations on inflammatory mediators. Chemical agents are being investigated for their contribution in cardiogenic shock. Nitric oxide production has also been thought to play a role in the development of cardiogenic shock and some studies indicate that inhibition of nitric oxide production may improve outcomes [89-91].

Thus, support systems in the form of mechanical devices and medications will have an increasing role in the future as part of our management strategies.

## CONCLUSION

Cardiogenic shock due to AMI continues to be the main cause of death in these patients. Immediate diagnosis and management is required. There are two strategies in treating cardiogenic shock: medical versus invasive. If institutions lack revascularization facilities, fibrinolytic therapy and IABP should be used while provisions are made for invasive treatment. However, current guidelines favor an invasive approach. Prognosis is established by the outcome of revascularization regardless of the procedure used, such as PCI or surgery [92]. Newer devices are being developed for mechanical support. Inhibitors of nitric oxide have also shown favorable outcomes. These newer therapies may help in decreasing the significant mortality of cardiogenic shock in the future.

## Figures and Tables

**Table 1:** Causes of Cardiogenic Shock

Acute Myocardial Infarction
Left ventricular dysfunction
Acute mitral regurgitation
Ventricular septal rupture
Right ventricular shock
Cardiac Tamponade
Cardiac Rupture

**Table 2:** Predisposing Factors for Cardiogenic Shock

Age
Sysolic blood pressure
Heart rate
Killip class
Diabetes
Anterior infarction
Previous infarction
Peripheral vascular disease
Reduced ejection fraction
Large infarctions
Cardiac power

**Table 3:** Clinical Signs of Cardiogenic Shock

Cyanosis
Rapid and/or faint pulses
Jugular venous distension
Cold extremities
Pulmonary rales
Distant heart sounds
Third and fourth heart sounds
Decreased urine output
Altered mental status

SBP=Systolic blood pressure

**Table 4:** Diagnostic Evaluation of Cardiogenic Shock

CBC
Electrolytes
Cardiac enzymes
Coagulation profile
ABG
CXR
ECG
Echocardiogram
Right heart catheterization

CBC=Complete blood count ABG=arterial blood gases CXR=chest x-ray ECG=elctrocardiogram
